# Web-based Gene Pathogenicity Analysis (WGPA): a web platform to interpret gene pathogenicity from personal genome data

**DOI:** 10.1093/bioinformatics/btv598

**Published:** 2015-10-21

**Authors:** Juan J. Diaz-Montana, Owen J.L. Rackham, Norberto Diaz-Diaz, Enrico Petretto

**Affiliations:** ^1^School of Engineering, Pablo de Olavide University, Seville, 41013 Spain and; ^2^Duke-NUS Graduate Medical School Singapore, Singapore, 169857 Singapore

## Abstract

**Summary:** As the volume of patient-specific genome sequences increases the focus of biomedical research is switching from the detection of disease-mutations to their interpretation. To this end a number of techniques have been developed that use mutation data collected within a population to predict whether individual genes are likely to be disease-causing or not. As both sequence data and associated analysis tools proliferate, it becomes increasingly difficult for the community to make sense of these data and their implications. Moreover, no single analysis tool is likely to capture all relevant genomic features that contribute to the gene’s pathogenicity. Here, we introduce Web-based Gene Pathogenicity Analysis (WGPA), a web-based tool to analyze genes impacted by mutations and rank them through the integration of existing prioritization tools, which assess different aspects of gene pathogenicity using population-level sequence data. Additionally, to explore the polygenic contribution of mutations to disease, WGPA implements gene set enrichment analysis to prioritize disease-causing genes and gene interaction networks, therefore providing a comprehensive annotation of personal genomes data in disease.

**Availability and implementation:** wgpa.systems-genetics.net

**Contact:** enrico.petretto@duke-nus.edu.sg

**Supplementary information:** Supplementary data are available at *Bioinformatics* online.

## 1 Motivation

With the growing volume of patient-specific sequences that is being generated there is an increasing need to annotate these data and distinguish possible disease causing mutations from benign mutations. To this end, a number of approaches have been developed to prioritize genes based on their predicted pathogenicity using whole-exome and whole-genome data. A recently introduced class of approaches use the pattern of functional sequence variation (i.e. rare and common mutations) observed in the human population (Petrovski *et al.*, 2013), the likelihood of observed mutations according to evolution (Rackham *et al.*, 2014) or statistical modelling of genes under selective constraint (Samocha *et al.*, 2014) to prioritize (rank) disease-causing genes from sets of genes impacted by mutations. Differently from sequence variant-level analysis (e.g. PolyPhen2 (Adzhubei *et al.*, 2013)), these methods specifically allow a *gene-level analysis* of pathogenicity, providing elegant, yet distinct schemes to evaluate the significance for individual genes in disease (Enns *et al.*, 2014; Shashi *et al.*, 2014). Here we provide an easy to use web-based tool (Web-based Gene Pathogenicity Analysis or WGPA) that integrates these methods for *gene-level* pathogenicity analysis (Petrovski *et al.*, 2013; Rackham *et al.*, 2014; Samocha *et al.*, 2014) as well as any future scoring system, therefore facilitating the assessment of the evidence supporting a role for a gene or variant in disease pathogenesis. Beyond single-gene analyses, WGPA provides a means to assess and test pathogenicity (using gene set enrichment analysis (Subramanian *et al.*, 2005)) for groups of genes of interest, look for mutations in the so called hot-zone using the gene level scores in conjunction with PolyPhen-2 (Adzhubei *et al.*, 2013) or FATHMM (Shihab *et al.*, 2013) and also to incorporate information from known gene interaction networks all within the same web based framework. Our platform will allow the scientific community to critically evaluate and interpret the large sets of mutation data from sequencing studies, aiding in the identification of genes and networks that play a critical role in disease aetiology.

## 2 Methods and implementation

### 2.1 Measures of genic intolerance

To date, only a few methods to predict pathogenicity at the gene level using sequence or population information alone are available: Residual variance intolerance score (RVIS) (Petrovski *et al.*, 2013), Evolutionary intolerance score (EvoTol) (Rackham *et al.*, 2014) and gene constraint scores (GCS) (Samocha *et al.*, 2014). The combination of these techniques with other analysis tools can provide a means to assess pathogenicity for sets of genes that have been found to be mutated in a disease, such as those identified by whole-exome and whole-genome sequencing. Here we provide a web-based tool that integrates in a single framework of analysis the following genic intolerance measures:

- RVIS identifies an intolerant gene as a gene containing a higher number of rare mutations than would be expected compared to other genes with a similar number of mutations.
- EvoTol identifies an intolerant gene as a gene containing an excess of mutations that, on the protein space, are not favoured by evolution as compared with other genes with the same number of mutations.
- GCS identifies excessively constrained genes using a statistical model which allow to rank genes based on their relative deficiency of functional variation.

### 2.2 Gene set enrichment analysis of gene pathogenicity

The methods described above provide gene-level scores for the identification of variants and genes that have a critical role in disease; these scores can be used to create ranked gene lists where individual highly intolerant (or constrained) genes can be prioritized. In order to integrate these scores over sets of genes, we provide a gene set enrichment analysis (GSEA) implementation (Subramanian *et al.*, 2005) that can be used with RVIS, EvoTol or GCS. Briefly, given a ranked list of genes (calculated genome-wide for each method described above) the GSEA tool tests if the genic intolerance scores of a subset of genes (provided by the user) occupy higher (or lower) positions in the ranked gene list than what it would be expected by chance. Gene set enrichment scores and significance level of the enrichment (*P*-value, False Discovery Rate (FDR), FWER *P*-value) are provided, using the GSEA output format developed by Broad Institute of MIT and Harvard (Subramanian *et al.*, 2005).

### 2.3 Interactome data

Genes that are mutated in disease do not operate in isolation, but as part of highly complex cellular and regulatory systems. A number of sources of gene interaction data are available, and here we use the STRING database (von Mering *et al.*, 2003), which provides several types of gene-gene interaction data. In order to remove less reliable interactions, we have filtered the STRING network to include only those interactions that have a STRING confidence score greater than 500 and are experimentally supported (Rackham *et al.*, 2014). The interaction data is used to display the pathogenicity scores for a set of genes on a network which, for instance, can be used to indentify genes that are both intolerant to mutation and network hubs.

### 2.4 Tools for annotating individual SNPs

In the development of RVIS the authors also defined the ‘hot-zone’ of mutation. This is a set of mutations that are both predicted to be damaging and also lie within genes that are predicted to be intolerant to mutation. In order to generalize this concept we have integrated both PolyPhen-2 and FATHMM, allowing for the hot-zone to be created as a combination these with of any of the three measures of intolerance.

### 2.5 Web interface

**Fig. 1.**
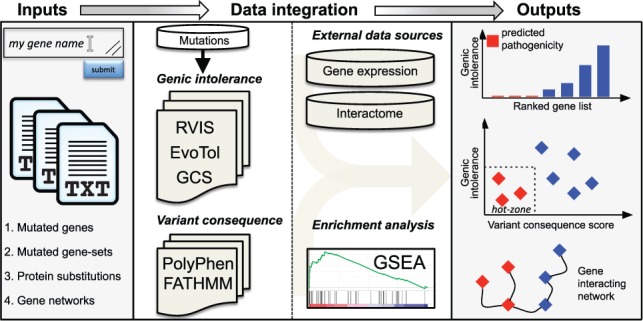
Schematic representation of the inputs, integrative data analyses component and associated outputs available through WGPA

In order to facilitate the annotation of personal genomes data with respect to disease pathogenesis, we have developed a unified web-based tool for pathogenicity analysis of individual genes, gene sets and gene interacting networks. To this aim, we developed an intuitive graphical user interface that will make the available prioritization methods (RVIS, EvoTol, GCS) and integrated analysis tools (GSEA, cell-type specificity, gene interacting networks) easy to access and use by the general scientific community. The type of input data, integrative analyses components and outputs are schematically summarized in Figure 1, and include the following inputs, analyses and outputs:

- **Inputs –** *Gene-Level:* manual data entry; gene list (*.txt); GRP, gene set (*.grp); GMX, gene matrix (*.gmx); GMT, gene matrix transposed (*.gmt); WGCNA, weighted gene co-expression network analysis output (*.wgcna); *Variant-Level:* manual data entry; list of protein substitutions (*.txt); list dbSNP identifiers (*.txt); *Network-Level:* manual data entry; list of gene identifiers for STRING (*.txt); list of gene pairs (*.txt)
- **Analyses –** RVIS, EvoTol (can be stratified by gene expression), GCS (user-selected); RVIS, EvoTol, GCS combined with variant-level consequence predictions (PolyPhen2 (Adzhubei *et al.*, 2013)) or FATHMM (Shihab *et al.*, 2013)); gene set enrichment analysis (for *Gene-Level* inputs)
- **Outputs –** genes ranked by their genic intolerance or constraint scores (graphical and table formats); GSEA results for gene sets (graphical and table formats); gene pathogenicity annotation using both the predicted ‘functionally damaging’ mutations and genic intolerance (or constraint) scores (to identify the so-called *hot-zone*, i.e. predicted both highly-intolerant and ‘functionally damaging’) (graphical and table formats); gene interaction network annotated according to RVIS, EvoTol or GCS allowing zooming out of a particular gene and visualizing its connections to other genes (graphical format).

## 3 Example

An example of where WGPA will be useful is to prioritize the set of genes with *de novo* mutations from trio sequencing projects. For instance in the Epi4K project Allen *et al.* (2013), trio sequencing was performed on epilepsy patients resulting in the identification of 329 *de novo* mutations impacting 176 different genes. By cross matching the RVIS, GCS and EvoTol scores and focusing on the genes from the top 25 percentile, we identify a set of 17 genes of interest (*ATP2B4, CHD4, DNM1, FLNA, FLNC, GABRA1, GABRB3, GNAO1, GRIN1, KCNQ2, MLL, MLL4, MYH6, SCN1A, SCN2A, SCN8A, WHSC1L1*, Supplementary Table S1). Using WGPA it was also possible to perform a GSEA of each of the measures of intolerance using the Epi4K mutated genes as the gene set of interest, and show that in each case the Epi4K mutated gene set is significantly enriched for predicted pathogenic genes (Supplementary Figure S1).

## Funding

Supported by The Duke-NUS Graduate Medical School Signatures Research Program (Program in Cardiovascular and Metabolic Disorders).

*Conflict of Interest*: none declared.

## Supplementary Material

Supplementary Data
