# Supplementary material for: Web-based Gene Pathogenicity Analysis (WGPA): a web platform to interpret gene pathogenicity from personal genome data
Source: Bioinformatics. 2015 Oct 21;32(4):635–7. doi: 10.1093/bioinformatics/btv598 (PMC4743624; doi:10.1093/bioinformatics/btv598)
Supplement: Supplementary Data [file supp_32_4_635__index.html]

Web-based Gene Pathogenicity Analysis (WGPA): a web platform to interpret gene pathogenicity from personal genome data — Supplementary Data 

# Web-based Gene Pathogenicity Analysis (WGPA): a web platform to interpret gene pathogenicity from personal genome data

## Supplementary Data

files

- Supplementary Data - pdf file
- Supplementary Data - pdf file
