# Supplementary material for: Web-based Gene Pathogenicity Analysis (WGPA): a web platform to interpret gene pathogenicity from personal genome data
Source: Bioinformatics. 2015 Oct 21;32(4):635–7. doi: 10.1093/bioinformatics/btv598 (PMC4743624; doi:10.1093/bioinformatics/btv598)
Supplement: Supplementary Data [file supp_btv598_SupplementaryFigure1.pdf]

Supplementary Figure 1: The set of genes containing *de novo* mutations in epilepsy (Allen *et al*. De novo mutations in epileptic encephalopathies. *Nature* 2013, 501(7466), 217-221) is used as a "gene set" and tested against all genes in the genome, which have been ranked according to their gene pathogenicity as predicted by Gene Constraint Score (GCS), RVIS and EvoTol. The graphical output of GSEA results generated by WGPA for the Epi4K "gene set" show that for each method (GCS, RVIS or EvoTol) there is a significant and consistent enrichment for pathogenic genes. Therefore, this WGPA analysis of the Epi4K data allowed to identify a sub set of genes that are predicted to be highly pathogenic, which can be used to inform future studies. The complete set of genes ranked according to their pathogenicity by GCS, RVIS and EvolTol, and the set of genes that are predicted to be highly pathogenic by all methods are reported in Supplementary Table 1.

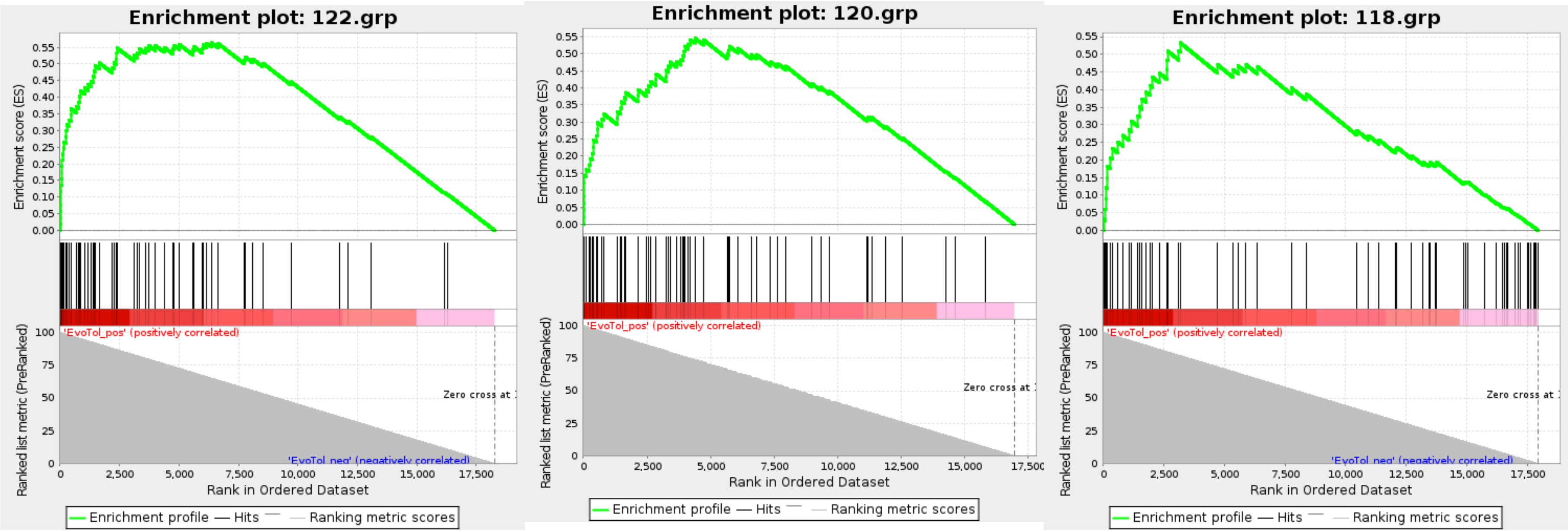

GCS *RVIS* *EvoTol*
