# Supplementary material for: Web-based Gene Pathogenicity Analysis (WGPA): a web platform to interpret gene pathogenicity from personal genome data
Source: Bioinformatics. 2015 Oct 21;32(4):635–7. doi: 10.1093/bioinformatics/btv598 (PMC4743624; doi:10.1093/bioinformatics/btv598)
Supplement: Supplementary Data [file supp_btv598_SupplementaryTable1.pdf]

Supplementary Table 1. Pathogenicity analysis with respect to the *de novo* mutations identified in the Epi4K project (Allen et al. (2013) De novo mutations in epileptic encephalopathies. Nature, 501, 217–221). For each method, we report the genes within the 25th percentile and highlight a subset of 17 genes identified in the Epi4K study that are predicted to be pathogenic by all methods (RVIS, Gene Constraint Score and EvoTol).

Gene symbol	RVIS %ile	Gene Constraint %ile	EvoTol score %ile	Epi4K
ATP2B4	1.56	11.51	20.48	YES
CHD4	6.36	2.82	0.17	YES
DNM1	14.79	19.54	1.43	YES
FLNA	0.91	0.44	0.29	YES
FLNC	0.59	0.29	2.52	YES
GABRA1	11.32	24.00	9.02	YES
GABRB3	12.85	22.36	7.61	YES
GNAO1	17.72	13.94	6.48	YES
GRIN1	10.72	6.72	0.20	YES
KCNQ2	0.51	15.86	1.34	YES
MLL	2.00	0.41	0.24	YES
MLL4	4.23	1.12	0.73	YES
MYH6	2.40	0.66	24.09	YES
SCN1A	0.04	4.03	2.50	YES
SCN2A	0.93	1.77	0.42	YES
SCN8A	4.46	2.34	0.13	YES
WHSC1L1	9.46	19.93	3.38	YES
ABCA2	2.19	0.12	0.21	NO
ABCC1	5.35	11.29	11.02	NO
ABCC5	10.46	1.82	3.03	NO
ABCC8	1.37	1.57	7.59	NO
ABCC9	1.24	1.82	6.91	NO
ABCD1	5.42	20.54	12.82	NO
ABCD3	9.11	16.44	12.29	NO
ACACA	4.47	2.96	0.15	NO
ACLY	15.91	13.10	3.80	NO
ACTB	3.43	15.62	1.71	NO
ACTG1	6.20	1.91	2.65	NO
ACVR2B	15.79	12.88	16.34	NO
ADAR	16.35	16.71	5.54	NO
ADARB1	7.31	12.33	2.16	NO
ADCY3	23.31	1.42	5.47	NO
ADCY6	14.46	5.90	8.75	NO
ADCY9	4.66	2.28	6.63	NO
AFG3L2	9.04	17.91	10.18	NO
ALOX5	14.72	9.21	24.89	NO
ALS2	18.87	3.89	19.49	NO

AMPD2	2.79	3.79	2.80	NO
ANAPC5	13.56	19.80	8.70	NO
APP	0.16	8.80	10.36	NO
AQR	9.04	8.14	4.12	NO
AR	1.70	17.31	19.92	NO
ARHGEF1	22.64	2.07	22.81	NO
ASH1L	1.99	2.22	1.64	NO
ATAD2	5.89	9.75	16.66	NO
ATP13A1	10.09	5.34	3.39	NO
ATP13A2	1.16	6.10	24.55	NO
ATP13A3	12.61	8.95	22.48	NO
ATP1A1	3.89	8.37	0.61	NO
ATP1A2	9.03	4.43	1.04	NO
ATP1A3	15.59	3.37	0.26	NO
ATP2A1	2.29	4.27	10.02	NO
ATP2A2	6.18	3.04	2.09	NO
ATP2A3	0.20	3.74	23.43	NO
ATP2B1	9.32	7.94	1.48	NO
ATP2B2	4.75	1.89	0.77	NO
ATP2B3	3.10	2.68	6.61	NO
ATP4A	6.39	8.99	4.38	NO
ATP6V0A1	2.20	15.20	9.15	NO
ATP8A1	10.04	16.68	11.85	NO
ATP8A2	24.56	6.89	13.61	NO
ATP9A	20.73	2.80	1.09	NO
ATRX	0.85	9.75	2.47	NO
BAIAP2L2	18.41	24.19	19.97	NO
BANP	22.40	11.77	10.14	NO
BARHL2	17.21	19.31	17.65	NO
BEGAIN	15.11	18.06	7.16	NO
BEND3	10.95	7.54	4.78	NO
BRAF	4.97	17.75	4.81	NO
BTAF1	13.36	2.47	4.96	NO
C10orf137	12.66	13.15	17.00	NO
C20orf112	19.11	7.66	11.62	NO
CACNA1C	0.03	1.57	0.18	NO
CACNA1D	0.57	0.32	2.01	NO
CACNA1E	7.05	0.71	0.83	NO
CACNA1G	0.06	1.11	0.76	NO
CACNA1I	1.97	11.55	0.38	NO
CACNB1	12.68	19.73	11.47	NO
CASZ1	0.59	1.06	6.47	NO
CD14	7.67	14.40	24.96	NO
CHD1	5.21	12.05	7.26	NO
CHD2	10.98	2.37	1.15	NO
CHD3	0.96	0.97	0.64	NO

CHD5	3.72	0.49	0.43	NO
CHD7	10.66	0.57	4.28	NO
CHD8	6.13	1.18	1.34	NO
CHD9	5.75	1.40	3.87	NO
CHRNB2	6.86	14.97	11.92	NO
CHRNE	11.38	10.03	3.75	NO
CIC	2.92	0.38	20.56	NO
CLCN3	5.16	18.59	5.81	NO
CLCN4	21.04	7.05	2.40	NO
CLCN6	5.89	3.17	20.10	NO
CLCN7	5.39	2.82	20.19	NO
CLPTM1	7.68	6.27	16.29	NO
CNNM4	24.22	6.23	16.12	NO
CNTNAP1	19.29	1.21	2.34	NO
COBRA1	18.42	4.56	14.97	NO
COL11A2	0.62	7.62	7.53	NO
COL1A1	0.29	2.26	3.77	NO
COL1A2	0.28	6.36	16.32	NO
COL2A1	0.88	2.21	10.93	NO
COL4A1	2.39	0.62	18.55	NO
COMP	6.89	22.65	5.59	NO
CPSF7	12.41	17.03	23.96	NO
CPT1A	2.07	22.78	18.84	NO
CPT1C	2.24	14.78	11.65	NO
CREBBP	0.55	1.54	1.28	NO
CSDE1	2.62	8.32	12.68	NO
CTBP1	17.65	13.94	7.38	NO
CYLD	11.17	9.47	5.59	NO
DAAM1	15.80	3.25	20.58	NO
DAXX	3.30	11.88	24.49	NO
DCAF15	11.98	18.90	24.92	NO
DCTN1	1.09	10.58	15.95	NO
DDR1	1.38	5.31	18.62	NO
DDR2	3.28	13.05	22.41	NO
DGKD	20.33	1.16	8.60	NO
DGKH	18.66	4.92	17.88	NO
DHCR24	7.37	11.77	15.77	NO
DLG2	13.83	3.04	12.94	NO
DLL1	7.29	6.89	19.09	NO
DLL4	22.03	4.56	8.40	NO
DMAP1	3.44	13.45	17.23	NO
DNM1L	5.12	8.75	12.37	NO
DNM2	9.80	4.71	3.27	NO
DNM3	16.34	6.72	5.12	NO
DNMT1	19.67	3.33	0.67	NO
DNMT3A	3.08	6.48	2.39	NO

DNMT3B	0.53	2.54	15.79	NO
DPF2	23.25	24.00	7.71	NO
DPYSL2	16.67	13.45	6.30	NO
DPYSL5	10.73	22.51	7.26	NO
DSP	6.16	1.32	24.80	NO
DTNA	11.30	21.65	22.01	NO
ECE1	2.02	15.32	24.00	NO
EDC4	8.02	4.00	3.14	NO
EGFR	10.12	1.44	19.76	NO
EHD4	22.53	13.58	17.05	NO
EIF3D	15.62	19.31	5.51	NO
EIF4ENIF1	1.14	8.90	22.71	NO
EP300	1.33	0.26	7.23	NO
EP400	0.57	0.22	2.02	NO
EPB41L1	3.49	14.15	13.17	NO
EPC1	18.47	17.40	13.22	NO
EPHB6	24.60	4.00	19.28	NO
ERBB2	14.25	18.28	6.19	NO
ERBB4	17.31	1.79	24.45	NO
ESRRG	3.95	19.31	11.85	NO
EWSR1	4.18	12.88	9.98	NO
EXOC1	1.73	16.53	22.33	NO
EXOC7	0.71	9.61	17.63	NO
EXT1	9.69	6.43	20.81	NO
EXTL3	6.59	2.24	17.35	NO
EZH1	20.46	24.19	3.21	NO
EZH2	12.83	20.54	2.61	NO
F2	8.93	14.57	18.90	NO
F8	0.40	13.77	15.06	NO
FBN1	0.35	0.64	1.88	NO
FBXO18	2.04	1.68	14.23	NO
FGFR1	0.71	4.58	6.33	NO
FGFR3	1.35	1.64	23.86	NO
FGR	17.25	10.30	14.19	NO
FIP1L1	23.07	18.44	13.16	NO
FLNB	0.04	3.94	16.07	NO
FMNL3	15.46	8.10	5.19	NO
FOXO3	7.21	22.36	2.36	NO
FOXP2	9.17	11.68	12.41	NO
FOXP4	6.75	9.27	22.06	NO
FRMD4A	7.41	3.17	8.50	NO
FTSJD2	14.02	7.11	6.57	NO
FURIN	23.60	12.57	6.50	NO
FYN	14.50	20.54	13.67	NO
FZD1	22.00	9.90	3.00	NO
FZD7	17.88	23.43	1.45	NO

GABBR2	23.20	5.08	1.93	NO
GABRB2	23.27	14.97	17.73	NO
GABRD	17.05	21.65	9.10	NO
GANAB	1.71	16.02	11.54	NO
GAPVD1	7.00	10.95	12.13	NO
GATA3	8.48	16.36	5.92	NO
GGNBP2	11.54	9.47	19.66	NO
GLG1	1.26	2.65	3.88	NO
GNA12	13.03	23.04	17.86	NO
GNAT1	14.16	17.75	7.73	NO
GNAZ	22.64	11.06	4.82	NO
GPS1	11.85	10.43	22.26	NO
GRIA1	21.01	5.79	3.75	NO
GRIA2	9.94	12.88	9.39	NO
GRIA4	8.36	7.05	7.99	NO
GRIK2	8.81	6.48	13.68	NO
GRIK3	23.38	2.68	11.32	NO
GRIK5	23.85	6.52	3.60	NO
GRM3	12.26	5.08	3.37	NO
GRM4	6.29	0.96	18.05	NO
GRM5	0.68	5.26	5.72	NO
GRM7	3.36	2.94	6.13	NO
GUCY1A2	12.95	7.66	14.39	NO
GUCY2D	4.98	4.51	19.89	NO
GUSB	2.80	11.88	11.80	NO
HCN1	14.96	13.33	5.49	NO
HECA	19.82	15.62	6.29	NO
HELZ	5.04	5.71	4.56	NO
HK1	2.28	5.64	1.63	NO
HK2	6.05	9.38	22.30	NO
HNF1B	3.95	20.26	12.96	NO
HNRNPUL1	12.31	14.08	1.97	NO
HPN	8.73	23.25	19.64	NO
HSD11B2	9.79	23.25	24.66	NO
HTRA3	15.03	11.77	23.37	NO
IGF1R	19.36	2.49	9.50	NO
ING1	21.67	21.56	19.97	NO
INO80	4.63	6.93	5.26	NO
INPPL1	4.36	2.84	17.61	NO
INSR	2.44	1.49	2.79	NO
INSRR	9.71	1.28	14.86	NO
INTS3	13.60	5.03	2.05	NO
INTS4	18.70	18.78	17.48	NO
INTS5	6.83	8.95	11.90	NO
INTS6	19.00	10.73	17.98	NO
IRF2	19.01	17.75	11.78	NO

IRF4	11.45	17.91	15.09	NO
ISYNA1	5.00	17.31	6.85	NO
ITGB1	0.92	5.94	22.53	NO
ITPR1	2.13	0.40	0.27	NO
ITPR2	5.50	2.78	1.59	NO
ITPR3	1.67	0.17	1.83	NO
JAG1	4.26	1.49	19.41	NO
JAG2	1.09	4.99	7.55	NO
JAK3	12.25	8.56	7.44	NO
JARID2	8.77	2.42	8.24	NO
KCNA1	11.97	19.54	4.67	NO
KCNA4	18.89	20.54	23.28	NO
KCNA6	23.97	21.20	1.25	NO
KCNB2	20.63	6.83	14.25	NO
KCNC1	10.81	15.62	1.06	NO
KCNC2	1.94	11.68	8.11	NO
KCND2	13.72	16.44	6.35	NO
KCND3	4.65	12.24	1.25	NO
KCNH1	4.21	7.15	9.38	NO
KCNH2	0.02	3.81	3.45	NO
KCNH3	5.70	10.56	3.23	NO
KCNH4	5.18	2.66	12.54	NO
KCNH5	2.25	3.81	3.52	NO
KCNJ2	3.96	18.44	20.36	NO
KCNJ4	15.60	10.30	1.65	NO
KCNMA1	1.62	2.65	0.84	NO
KCNN3	11.62	19.54	3.15	NO
KCNQ4	4.04	15.76	2.98	NO
KCNQ5	1.04	14.08	5.22	NO
KDM5A	10.18	13.79	15.72	NO
KDM5B	7.25	2.48	9.64	NO
KDM5C	14.91	3.50	1.03	NO
KIAA0430	2.55	3.45	11.29	NO
KIAA0664	21.47	1.33	1.85	NO
KIAA0907	22.43	18.44	17.39	NO
KIAA1468	24.94	5.08	4.74	NO
KIAA1598	20.41	24.00	15.47	NO
KIF26B	20.89	18.26	15.36	NO
KIF5A	24.84	4.88	2.82	NO
KIF5B	18.24	9.55	16.70	NO
KIT	3.44	15.36	19.81	NO
LGI1	19.11	14.40	21.81	NO
LIN54	3.89	20.54	20.61	NO
LIN9	14.04	22.36	21.96	NO
LMNA	0.07	9.96	5.80	NO
LMX1B	17.79	11.06	10.50	NO

LRP1	0.81	0.02	0.04	NO
LRP1B	0.42	1.23	20.70	NO
LRP4	1.72	4.69	5.92	NO
LRP5	1.24	0.24	6.90	NO
LRP6	3.07	2.57	19.45	NO
LTBP2	3.40	3.29	19.08	NO
LTBP3	5.20	9.61	2.55	NO
MATK	17.03	19.80	8.66	NO
MBD1	1.08	13.58	12.23	NO
MCRS1	6.76	23.25	19.53	NO
MEPCE	10.47	10.30	6.12	NO
MFSD2A	8.22	22.36	16.77	NO
MGA	1.56	17.48	16.17	NO
MGAT3	8.95	18.72	5.46	NO
MLL2	13.34	0.06	0.31	NO
MLL3	0.21	0.91	14.17	NO
MLLT1	15.14	17.31	8.65	NO
MNT	12.23	13.45	24.86	NO
MON1A	5.36	18.72	13.49	NO
MOV10	2.31	2.65	15.84	NO
MTM1	14.81	21.20	20.00	NO
MTMR4	4.83	19.93	13.83	NO
MYH11	0.40	0.47	17.55	NO
MYH3	3.87	3.23	7.89	NO
MYH7	2.21	0.28	0.39	NO
MYH9	8.18	1.75	1.08	NO
MYO10	7.54	2.97	19.64	NO
MYO15A	1.00	5.35	9.21	NO
MYO18A	4.14	2.90	5.54	NO
MYO1F	8.75	2.12	21.03	NO
MYO5A	9.51	4.15	3.54	NO
MYO9B	4.53	10.93	6.93	NO
NALCN	4.09	2.98	0.63	NO
NCDN	12.36	8.75	1.95	NO
NF1	6.59	0.47	0.63	NO
NF2	1.52	10.65	11.46	NO
NFRKB	1.47	0.76	14.27	NO
NIPBL	0.75	1.01	1.99	NO
NKRF	20.53	20.26	20.13	NO
NLRP3	0.13	9.38	12.72	NO
NOL4	13.72	15.76	7.59	NO
NOTCH1	4.56	0.33	1.40	NO
NOTCH2	3.77	2.15	4.93	NO
NOTCH3	2.34	5.04	6.01	NO
NPR2	17.76	7.71	2.15	NO
NR0B1	11.20	22.09	13.38	NO

NR2C2	11.44	10.37	13.99	NO
NR4A2	22.85	17.03	5.06	NO
NRP1	4.74	22.75	13.06	NO
NRP2	0.77	7.28	22.83	NO
NSD1	0.51	3.24	5.43	NO
NTRK2	10.13	22.65	15.54	NO
ODZ1	1.30	2.62	15.54	NO
ODZ3	6.04	0.36	3.83	NO
OLFM1	20.80	17.75	7.04	NO
PANK4	16.16	8.95	21.32	NO
PAPL	10.80	23.51	24.63	NO
PAPOLA	14.44	17.03	20.62	NO
PARP8	1.29	9.96	18.82	NO
PAX7	1.86	6.78	16.62	NO
PC	0.63	2.39	3.59	NO
PCIF1	9.52	11.28	13.72	NO
PCSK7	19.77	13.67	13.53	NO
PDS5A	18.84	15.20	1.11	NO
PDS5B	16.27	3.64	2.75	NO
PFKFB2	9.02	15.76	22.53	NO
PFKFB3	7.10	4.19	18.99	NO
PFKM	10.91	23.57	23.86	NO
PHF12	3.85	6.52	24.70	NO
PHKA1	0.99	12.46	23.34	NO
PIK3R1	21.01	13.45	17.22	NO
PLEKHH3	14.45	9.17	13.41	NO
POU2F2	17.66	20.26	17.54	NO
POU4F2	16.95	18.06	22.12	NO
PPARD	0.79	18.44	12.26	NO
PPP2R5B	13.26	20.26	20.84	NO
PRICKLE2	16.13	6.78	9.77	NO
PRKAG2	1.65	10.73	22.54	NO
PRMT5	17.05	19.54	7.39	NO
PRPF3	20.88	19.31	6.55	NO
PSMC3	15.83	23.04	14.45	NO
PSMC4	19.48	19.31	6.36	NO
PTCHD1	13.12	8.32	17.33	NO
PTPN9	24.87	19.54	22.55	NO
PTPRA	9.63	7.15	15.74	NO
QRICH1	7.88	23.25	3.69	NO
QTRT1	16.05	21.41	14.33	NO
RAF1	21.76	16.44	13.75	NO
RALGAPA1	3.49	5.71	5.91	NO
RALGAPB	4.45	9.32	9.58	NO
RARA	8.40	23.25	5.44	NO
RARB	14.24	22.09	12.51	NO

RASAL2	22.11	1.53	17.89	NO
RB1	5.72	15.12	22.32	NO
RBM14	14.74	14.57	5.34	NO
RBPJ	12.01	21.20	11.60	NO
RCC1	11.13	19.54	24.40	NO
RERE	20.13	0.78	3.84	NO
RET	1.78	3.00	12.84	NO
RNF10	7.44	18.14	18.43	NO
RNF220	11.05	16.44	20.88	NO
RNF8	14.10	20.26	20.34	NO
RNGTT	20.43	19.54	17.91	NO
RORA	0.61	9.09	18.30	NO
RPS2	20.04	19.31	7.47	NO
RSBN1	12.15	11.18	5.41	NO
RXRA	19.04	15.12	10.04	NO
RXRG	23.54	11.06	12.66	NO
RYR1	0.03	0.01	1.29	NO
RYR2	3.75	0.05	0.12	NO
RYR3	1.58	0.06	20.44	NO
SAMHD1	7.01	14.02	14.60	NO
SAP130	2.42	2.94	11.73	NO
SBF1	7.36	0.17	15.15	NO
SBNO1	17.17	12.01	8.02	NO
SCN3A	0.65	0.97	1.20	NO
SCN4A	1.49	13.68	7.15	NO
SCN5A	0.00	1.99	3.66	NO
SCO2	1.69	17.31	5.11	NO
SCP2	5.18	12.24	24.61	NO
SEC61A1	24.57	20.26	4.57	NO
SERPINH1	9.13	17.91	11.05	NO
SETBP1	13.12	6.61	9.96	NO
SETD1A	3.86	0.56	5.68	NO
SETD5	5.92	12.60	18.54	NO
SETDB1	2.11	3.81	4.66	NO
SF3B1	16.78	6.43	0.34	NO
SF3B2	9.21	7.11	6.50	NO
SH3BP5L	16.47	13.94	23.77	NO
SHPRH	16.40	7.58	14.84	NO
SLC12A2	5.55	4.88	19.77	NO
SLC12A5	4.98	4.45	2.73	NO
SLC12A6	1.08	6.51	3.79	NO
SLC20A1	9.22	22.36	14.56	NO
SLC20A2	7.72	13.67	11.62	NO
SLC22A12	6.08	6.23	18.14	NO
SLC25A25	22.05	24.46	19.14	NO
SLC2A1	21.27	7.66	11.43	NO

SLC45A1	20.45	1.67	22.56	NO
SLC4A5	9.84	3.31	18.13	NO
SLC5A2	4.87	10.89	10.38	NO
SLC5A3	14.96	15.86	21.25	NO
SLC7A1	16.82	5.60	8.55	NO
SLIT1	18.22	1.70	2.24	NO
SLTM	19.53	7.77	7.34	NO
SMARCA2	6.95	1.82	0.38	NO
SMARCA4	1.11	0.60	0.09	NO
SMARCA5	17.29	8.75	2.95	NO
SNED1	12.35	4.36	16.92	NO
SNW1	19.18	22.09	6.94	NO
SOS1	17.22	8.54	19.52	NO
SOX10	23.78	23.04	7.03	NO
SOX5	11.09	9.09	6.39	NO
SOX6	2.10	6.72	10.89	NO
SOX9	17.63	14.40	2.48	NO
SRCAP	0.86	0.15	3.35	NO
SRRT	6.83	3.05	4.30	NO
SSBP3	6.88	16.36	24.18	NO
STAT3	5.94	22.36	1.60	NO
STAT5A	19.13	16.53	3.72	NO
STAT5B	17.79	10.30	5.33	NO
STAT6	2.06	15.27	16.44	NO
STT3A	15.98	16.36	4.53	NO
STT3B	11.60	13.33	6.64	NO
SULF1	0.74	22.75	17.89	NO
SUPT16H	15.44	17.31	1.52	NO
SUPT5H	7.00	4.10	0.54	NO
SUPT6H	7.48	1.07	2.11	NO
SYK	6.97	13.45	6.40	NO
SYMPK	13.44	3.69	1.57	NO
TADA3	11.88	14.40	21.16	NO
TBX3	5.93	14.08	21.24	NO
THBS1	12.36	2.39	21.67	NO
THBS2	12.64	23.05	24.62	NO
THOC1	18.76	24.19	18.42	NO
THRA	3.41	19.31	3.29	NO
THRB	1.29	23.04	12.61	NO
TNIK	22.11	3.97	3.46	NO
TNPO3	15.22	5.94	9.99	NO
TP63	0.39	9.96	5.87	NO
TP73	3.27	2.44	21.54	NO
TPCN1	6.42	7.20	10.56	NO
TPP2	11.37	6.27	7.02	NO
TRAF6	18.92	20.26	17.79	NO

TRIM3	21.30	5.76	2.70	NO
TRIM71	21.14	6.43	2.03	NO
TRPC3	10.84	5.41	8.44	NO
TRPC6	10.46	5.08	20.73	NO
TSC1	1.30	7.83	5.55	NO
UBTF	13.96	6.17	2.24	NO
UCKL1	5.59	4.19	16.41	NO
UPF1	13.36	3.39	0.30	NO
VANGL2	16.42	7.94	16.58	NO
VASH1	19.70	22.09	19.90	NO
VAV2	19.76	2.55	23.76	NO
VIM	8.85	11.68	12.66	NO
VPS52	10.50	21.56	9.83	NO
WDR11	5.07	9.75	16.33	NO
WDR33	6.23	12.05	4.48	NO
WHSC1	0.89	4.48	14.14	NO
WIZ	15.92	12.46	4.14	NO
XKR4	22.42	11.68	5.90	NO
XKR6	23.12	11.68	3.95	NO
XPNPEP1	7.86	14.02	17.34	NO
XRCC6	16.56	15.62	10.20	NO
YME1L1	6.01	11.88	24.87	NO
ZAP70	4.62	6.17	5.46	NO
ZC3H14	1.18	16.63	21.55	NO
ZMYND8	2.58	3.98	10.74	NO
ZNFX1	2.75	24.22	16.16	NO
